# Clinical Neuropathology image 1-2018: Golgi silver staining, the black reaction 

**DOI:** 10.5414/NP301087

**Published:** 2017-12-18

**Authors:** Sara Mariotto, Marina Bentivoglio, Tiziana Cotrufo, Antonella Berzero, Salvatore Monaco, Paolo Mazzarello, Sergio Ferrari

**Affiliations:** 1 Department of Neuroscience, Biomedicine and Movement Sciences, University of Verona,; 2 University Museum, and; 3 Department of Nervous System Sciences and Behavior, University of Pavia, Italy; *On leave from the Department of Cell Biology, Physiology and Immunology, Faculty of Biology, University of Barcelona, Spain.

**Keywords:** Camillo Golgi, neuronal staining, history of neuroscience, hippocampus

## Abstract

No Abstract available.

We here present an image from the rare original slides from Camillo Golgi (Figure 1), which are kept at the Golgi Museum and at the Museum for the History of the University of Pavia (Pavia, Italy). 

Camillo Golgi (1843 – 1926), Professor of General Pathology and Histology at the University of Pavia, provided seminal scientific contributions, including in 1898 the discovery of the cell organelle named after him Golgi apparatus [1, 2]. Before then, Golgi had discovered the silver staining technique, known as “the black reaction” (*reazione nera*), published in 1873 [3]. The procedure, based on the fixation of nervous tissue blocks in potassium dichromate and impregnation in a solution of silver nitrate, results in black deposits that fill the neurons in their entirety (somata, dendrites, and axons). By staining serendipitously a limited number of cells, the Golgi impregnation allowed to visualize for the first time neurons (together with glial cells) with the full extent of their processes (Figure 1). 

This was a breakthrough that opened a new era in neuroscience, neurology, and neuropathology. Golgi documented his pioneer observations with detailed descriptions and drawings [4], reporting novel findings, such as the free ending of dendritic arborizations, axons as the output elements, and the occurrence of axonal branching, as constant features of nerve cells. To account for the complexity of nervous transmission in the brain, Golgi hypothesized a reticular system of interactions among axons in continuity. More than a decade later, this “reticular” organization, which Golgi ascribed to a “diffuse nervous network”, was fiercely opposed by the “neuron theory” stating that axons are in contiguity and not in continuity. This was largely due to the monumental work of Santiago Ramón y Cajal (1852 – 1934) on the structure of the nervous system based on the Golgi impregnation. The neuron doctrine became the founding cellular paradigm of nerve cell structure and function. 

Golgi and Cajal shared the Nobel Prize in Physiology and Medicine “in recognition of their work on the structure of the nervous system” in 1906, when they presented in their lectures the contrasting theories. 

The revelatory power of the black reaction (Figure 1) was the turning point of 19^th^ century neuroanatomy, and this staining was one of the most powerful techniques for the foundation of modern neuroscience. 

## Funding 

None. 

## Conflict of interest 

The authors report no conflict of interest. 

**Figure 1.**
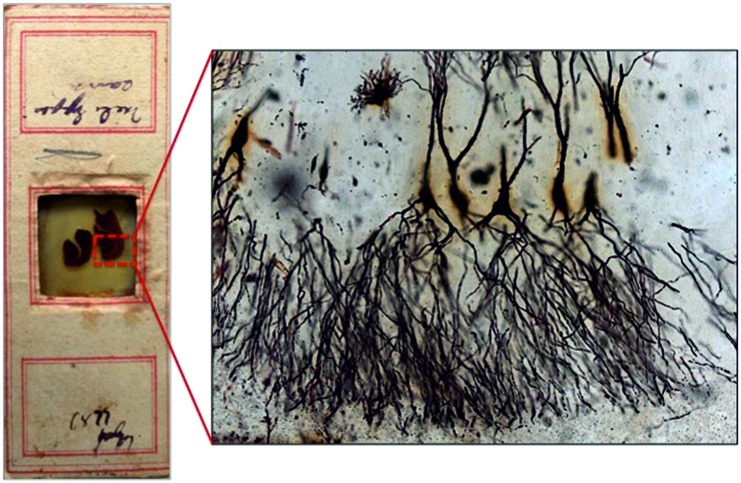
Original slide of Camillo Golgi, with his signature, stained in 1899 (left). Image from a Golgi’s slide of hippocampal neurons impregnated by the black reaction (right).
